# Tau Oligomers: Cytotoxicity, Propagation, and Mitochondrial Damage

**DOI:** 10.3389/fnagi.2017.00083

**Published:** 2017-04-04

**Authors:** Scott S. Shafiei, Marcos J. Guerrero-Muñoz, Diana L. Castillo-Carranza

**Affiliations:** ^1^Department of Neurology, Neuroscience and Cell Biology, University of Texas Medical BranchGalveston, TX, USA; ^2^Department of Chemical Engineering, Hampton UniversityHampton, VA, USA; ^3^Minority Men's Health, Hampton UniversityHampton, VA, USA

**Keywords:** tauopathy, tau oligomers, tau secretion, propagation, mitochondrial dysfunction

## Abstract

Aging has long been considered as the main risk factor for several neurodegenerative disorders including a large group of diseases known as tauopathies. Even though neurofibrillary tangles (NFTs) have been examined as the main histopathological hallmark, they do not seem to play a role as the toxic entities leading to disease. Recent studies suggest that an intermediate form of tau, prior to NFT formation, the tau oligomer, is the true toxic species. However, the mechanisms by which tau oligomers trigger neurodegeneration remain unknown. This review summarizes recent findings regarding the role of tau oligomers in disease, including release from cells, propagation from affected to unaffected brain regions, uptake into cells, and toxicity via mitochondrial dysfunction. A greater understanding of tauopathies may lead to future advancements in regards to prevention and treatment.

## Introduction

Neurodegenerative diseases are a leading cause of death and disability affecting millions of elderly. As life expectancies rise, growing numbers of elderly become affected. This emerges as a major global issue as there are currently no adequate remedies for these diseases. A large group of these diseases, which are related to tau, a microtubule-associated protein, are known as tauopathies. Most notably, tauopathies include Alzheimer's disease (AD), progressive supranuclear palsy (PSP), Pick's disease, frontotemporal dementia (FTD), corticobasal degeneration, and variants of Parkinson's disease (PD) and Lewy body dementia (LBD; Goedert et al., 2000; Hutton, 2000; Spillantini et al., 2000). These share a common histopathological hallmark known as neurofibrillary tangles (NFTs) that consist of an accumulation of fibrillar tau deposits initially produced from tau protein aggregation (Ballatore et al., 2007).

While functional tau is an unfolded monomeric protein that stabilizes microtubules, regulates neurite growth, and monitors axonal transport of organelles (Medina and Avila, 2014), dysfunctional tau acquires a new toxic function.

## Toxicity of tau oligomers vs. NFTs

NFTs, although considered a histopathological hallmark in tauopathies, do not appear to be the main toxic entities leading to disease (Gerson et al., 2014b). In AD, tau pathology and neuronal cell loss coincide in the same brain regions, and as brain dysfunction progresses, NFTs are found in greater anatomical distributions (Ihara, 2001). However, the role of NFTs in the progression of the disease is poorly understood. Compared to non-demented controls, AD brains exhibit up to 50% of neuronal loss in the cortex, exceeding the number of NFTs (Gómez-Isla et al., 1997). In addition, neurons containing NFTs are functionally intact *in vivo* (Kuchibhotla et al., 2014) and have been found in brains of cognitively normal individuals. Further, intra-neuronal NFTs do not affect post-synaptic function and signaling cascades responsible for long-term synaptic plasticity in tauopathy mice overexpressing P301L mutant tau (Rudinskiy et al., 2014), suggesting that synaptic deficits cannot be attributed to NFTs.

While evidence has linked FTD with parkinsonism in patients to tau mutations on chromosome 17 (FTDP-17), implying that tau dysfunction alone can cause neurodegeneration (Reed et al., 2001), studies in animal models have shown that overexpression of tau can lead to cell death (Lee et al., 2001; Tanemura et al., 2001, 2002; Tatebayashi et al., 2002) and exhibit behavioral abnormalities and synaptic dysfunction without the presence of NFTs (Wittmann et al., 2001; Andorfer et al., 2003; Santacruz et al., 2005; Spires et al., 2006; Berger et al., 2007; Yoshiyama et al., 2007; Cowan et al., 2010). Others have noted neuronal loss without NFT presence in a *Drosophila* model overexpressing tau (Wittmann et al., 2001). These studies provide evidence that progressive tau accumulation in neurodegeneration may not require NFT formation (Maeda et al., 2006). Indeed, reducing tau overexpression in mutant tau transgenic mice decreases neuronal cell loss even though NFTs continue to form (Santacruz et al., 2005). This indicates that NFT formation is not essential for neuronal loss.

While evidence indicates that these deposits are not toxic, many studies suggest that the tau oligomer, an intermediate entity, is likely responsible for disease onset. Hyper-phosphorylated tau assembles into small aggregates known as tau oligomers in route of NFT formation. As hyper-phosphorylated tau dislodges from microtubules, its affinity for other tau monomers leads individual tau to bind each other, forming oligomeric tau, a detergent-soluble aggregate. These tau oligomers potentiate neuronal damage, leading to neurodegeneration and traumatic brain injury (Hawkins et al., 2013; Gerson et al., 2014a, 2016; Sengupta et al., 2015). Moreover, they have been implicated in synaptic loss as shown in studies of wild-type human tau transgenic mice (Spires et al., 2006; Berger et al., 2007; Clavaguera et al., 2013). When the oligomer lengthens, it adapts a β-sheet structure and transforms into a detergent-insoluble aggregate with granular appearance under Atomic Force Microscopy (AFM). As these granular tau oligomers fuse together, they form tau fibrils, which ultimately form NFTs (Takashima, 2013). These steps hint that tau oligomers may be involved in neuronal dysfunction prior to NFT formation (Maeda et al., 2006).

The onset of clinical symptoms in AD and PSP brains correlate with elevated levels of tau oligomer (Maeda et al., 2006, 2007; Patterson et al., 2011; Lasagna-Reeves et al., 2012b; Gerson et al., 2014a). When tau oligomers, rather than tau monomers or fibrils, are injected into the brain of wild-type mice, cognitive, synaptic, and mitochondrial abnormalities follow (Lasagna-Reeves et al., 2011; Castillo-Carranza et al., 2014b). Additionally, studies have discovered that aggregated tau inhibits fast axonal transport in the anterograde direction at all physiological tau levels, whereas tau monomers have had no effect in either direction (LaPointe et al., 2009; Morfini et al., 2009). This suggests that monomers are not the toxic entity either. Most noteworthy, tau oligomers induce endogenous tau to misfold and propagate from affected to unaffected brain regions in mice, whereas fibrils do not (Lasagna-Reeves et al., 2012a,b; Wu et al., 2013). This indicates that tauopathies progress via a prion-like mechanism dependent upon tau oligomers (Gerson and Kayed, 2013; Castillo-Carranza et al., 2014b). With this concept, tau may be able to translocate between neurons and augment toxic tau components; in fact, evidence suggests probability of tau oligomer propagation between synaptically connected neurons (Gendreau and Hall, 2013; Pooler et al., 2013b). If true, then pathology begins in a small area and becomes symptomatic as it spreads to other areas of the brain (Medina and Avila, 2014). Studies show that tau pathology progresses from the entorhinal cortex to the hippocampus, eventually leading to the limbic and association cortex; this progression explains the individuals clinical cognitive status (Nelson et al., 2012). Further, mice injected with tau oligomers in the proximity of the hippocampus experienced immediate memory impairment (Lasagna-Reeves et al., 2011). These studies demonstrate that tau oligomers may be the toxic entities responsible for neurodegeneration in tauopathies (Ward et al., 2012).

## Cellular tau secretion and propagation

Tau predominantly presents as an axonal cytoplasmic protein, however evidence has shown tau at the pre- and post-synapse in human brains (Tai et al., 2012) as well as at the post-synapse in mouse brains (Ittner et al., 2010). Further, tau directly interacts with synaptic proteins, including the NMDA receptor (Ittner et al., 2010; Mondragón-Rodríguez et al., 2012). This hints that tau plays a role in monitoring intracellular signaling pathways (Pooler and Hanger, 2010). Related evidence indicates that synaptic activity leads to tau monomer release (Pooler et al., 2013a; Yamada et al., 2014). Yet whether the movement of aggregates across the synapse occurs easily from nearby cells taking up released aggregated material at the axon terminal or whether the movement depends on activity is unknown.

Tau is also present outside the cell in brain fluids, including cerebrospinal fluid. In AD, the quantity of tau identified in the CSF increases with disease progression (Hampel et al., 2010). However, the mechanism of tau propagation from the brain to the CSF remains elusive. Recently, tau was discovered in the interstitial fluid of awake, wild-type mice, suggesting its release by neurons in the absence of neurodegeneration (Yamada et al., 2011). This evidence suggests that tau secretion is an active neuronal process separate from cell death (Saman et al., 2012; Pooler et al., 2013a).

In AD, misfolded and hyper-phosphorylated tau concentrates in various components of the neuron: dendrites, cell body, and axons (Avila et al., 2004). Hence, propagation of disease may depend on transport within neurons. Further, transgenic mouse lines expressing human tau aggregates in the entorhinal cortex have shown that tau is mislocalized from axons to cell bodies and dendrites as the mice age (Pooler et al., 2013b). Nevertheless, given that tau is detected in both axons and dendrites, it is possible that either region may be involved in its secretion (Pooler et al., 2013b). Extracellular tau is implicated as the primary agent during propagation of neurofibrillary lesions and spreading of tau toxicity (Medina and Avila, 2014). In trans-synaptic propagation, tau can be released and taken up by a synaptically-connected neuron (Clavaguera et al., 2009; Dujardin et al., 2014b; Dennissen et al., 2016). A recent study showed that neuronal networks facilitate cell-to-cell transfer of tau via synapses; using a microfluidic device they demonstrated that decreasing synaptic connections weakens tau transfer and the subsequent aggregation on the acceptor cell (Calafate et al., 2015).

Determining the mechanism behind the propagation of misfolded tau protein from one cell to another is currently of great significance in research. In AD, tau pathology has been found to spread from the transentorhinal cortex to the neocortex in a sequential pathway. This prion-like spreading of tau may occur throughout neuronal connections. However, the method of tau oligomer release via the cell and its spread is still unknown.

The prion concept suggests that a protein can be transformed into a disease-causing form when in contact with a pathogenic protein “seed.” The mechanism for the transformation is not well-understood; however, it includes templated conformational change (Telling et al., 1996) and is demonstrated to propagate through neural networks. Thus, the prion hypothesis serves as a useful model when testing ideas regarding propagation of protein pathology.

In trans-cellular propagation, tau aggregates escape from afflicted neurons into the extracellular space prior to entering adjacent or synaptically-connected cells. This suggests that extracellular tau may be susceptible to antibody-mediated therapies. According to this model, misfolded tau is released into the extracellular space and then gains entry into adjacent or synaptically-connected cells to trigger further aggregate formation via templated conformational change.

## Exosomes and ectosomes as a mechanism of tau spreading

Recently, more evidence implies that the secretion of tau occurs through unconventional cellular pathways via vesicles known as exosomes (Saman et al., 2012) and ectosomes (Dujardin et al., 2014a).

Exosomes are small membranous vesicles ranging from 30 to 100 nm, which are secreted from most cell types, including neurons. Exosomes have been identified in several body fluids (Witwer et al., 2013; Khalyfa and Gozal, 2014). They are made by the endocytosis of molecules and can assist in spreading pathology. Once taken up by a cell, the molecules inside the exosomes are either recycled to the plasma membrane or transported to multivesicular bodies (MVBs; Dujardin et al., 2014a). The fusion of MVBs with the plasma membrane results in exosomal release (Mathivanan et al., 2010). AD brain samples contain exosomal proteins within amyloid plaques hinting that exosomes play part in disease pathology (Rajendran et al., 2006). Tau, like other amyloidogenic proteins, may be secreted and spread via exosomal vesicles (Danzer et al., 2012; Asai et al., 2015). In support, tau associated with exosomes and phosphorylated at Thr-181 (AT270+ tau) has been identified in human CSF samples of AD patients. More recently, patients affected with FTD and AD, were found to have high levels of total tau and phosphorylated tau (p-T181 and p-S396; Saman et al., 2012). Further, peripheral exosomes extracted from AD cases, propagate tau pathology in the brain of normal mice (Winston et al., 2016). It was recently shown that microglial cells may facilitate tau spreading via exosomes. The authors speculated that microglia phagocytose tau-containing neurons or synapses and secrete tau protein via exosomes (Asai et al., 2015). Further investigation is needed to determine if predominant tau aggregates are released via this unconventional pathway.

**Figure 1:**
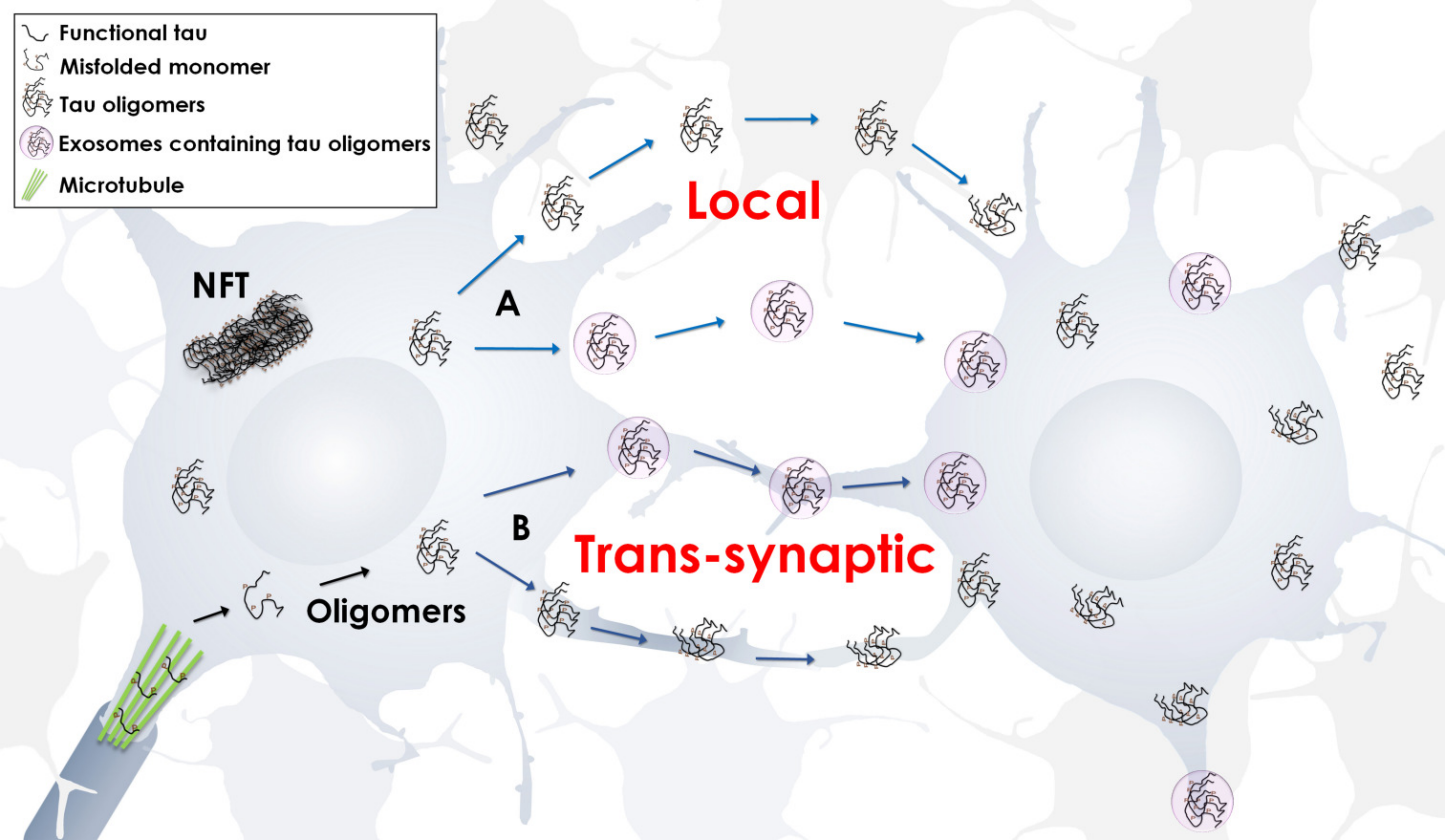
**Propagation of tau oligomers**. Schematic representation of free or exosomal tau oligomers release, local or trans-synaptic. In local transmission, tau oligomers are released from one neuron and taken up by another neuron in the vicinity. In trans-synaptic transmission, tau oligomers and/or exosomes containing tau oligomers are passed across the synapse of two neighboring neurons.

Exosomes are not the only vesicles that may spread pathology. Ectosomes are also extracellular vesicles, but range from 50 to 1,000 nm. They directly shed from cells by budding from the plasma membrane (Piccin et al., 2007; Cocucci et al., 2009; Théry et al., 2009; Davizon et al., 2010). Ectosomes are contenders in secreting tau protein since they are released via cell membrane activation by fluctuating intracellular levels of calcium, inflammatory molecules, or oxidative stress (Piccin et al., 2007; Doeuvre et al., 2009). Significantly, evidence suggests that tau secretion is partly mediated by ectosomal vesicles and that pathological tau accumulation in cells leads to a deviation toward tau secretion by exosomal vesicles (Dujardin et al., 2014a). These studies provide further evidence that extracellular vesicles play an important role in disease pathogenesis (Figure 1).

## Cellular uptake of tau oligomers

**Figure 2:**
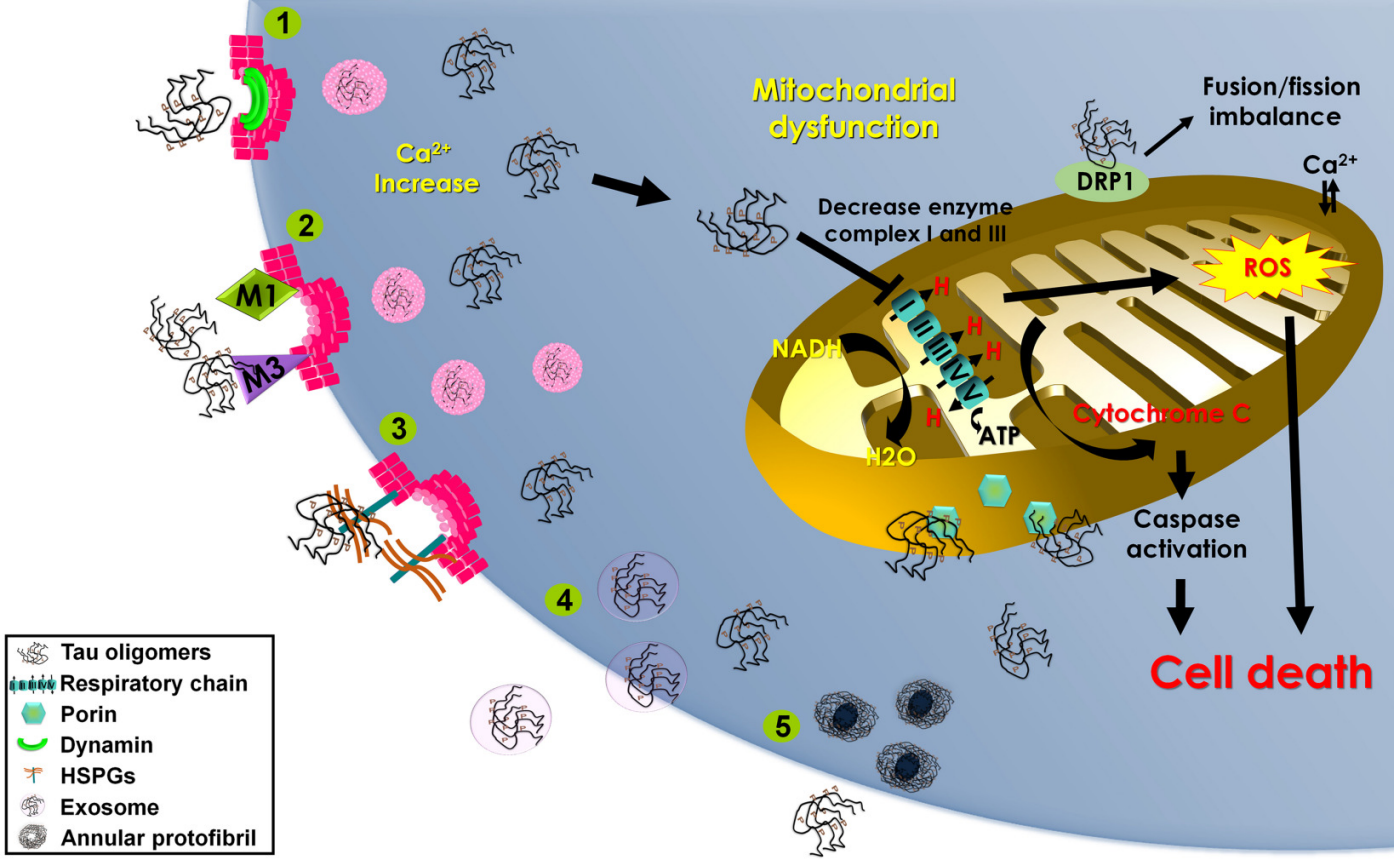
**Mechanisms of tau oligomer internalization and mitochondrial damage**. Schematic representation of the proposed mechanisms contributing to tau oligomer internalization that may lead to mitochondrial dysfunction and cell death. Methods of tau oligomer internalization include (1) dynamin-driven endocytosis; (2) muscarinic (M1 and M3) receptor-mediated endocytosis; (3) proteoglycan-mediated macropinocytosis (HSPGs); (4) exosomes; and (5) annular protofibrils. Internalized tau oligomers interfere with the mitochondrial respiratory chain, inducing cytochrome c release and stimulating reactive oxygen species (ROS) production. Tau oligomers induce mitochondrial fusion/fission imbalance by binding with dynamin-related protein 1 (DRP1). Tau oligomers interact with the outer membrane porin protein.

Quite a few mechanisms involving tau uptake have been proposed. These include (1) cell internalization of soluble, uncoated tau via receptor-mediated endocytosis (Gómez-Ramos et al., 2009), (2) dynamin-driven endocytosis of non-fibrillar, soluble tau aggregates (Wu et al., 2013), and (3) actin-dependent, proteoglycan-mediated macropinocytosis (Holmes et al., 2013; Figure 2).

## Receptor-mediated endocytosis

Tau may be endocytosed, promoting an increase in intracellular calcium that results in neuronal death. In the former theory, endocytosed tau may interact with various cellular products, including tau itself, and can be secreted, uncoated or in a membrane vesicle. Such secreted vesicles may interact with other cells and be endocytosed in an unspecific way. In the latter theory, during the secretion, vesicles and the cell membrane can be fused and uncoated tau protein can be released to the extracellular space (Clavaguera et al., 2009; Iba et al., 2013).

Tau can interact with muscarinic receptors; more specifically, M1 and M3 receptors have approximately a 10-fold higher affinity for tau than acetylcholine. Overstimulation with tau (as opposed to acetylcholine) does not desensitize the muscarinic receptors present on neurons of the hippocampus; hence, a repeat stimulus via tau increases intracellular calcium every time, thus altering intracellular calcium homeostasis and the following hyper-phosphorylation and misfolding of tau. Coupled with the fact that tau persists in the extracellular environment for a longer time than acetylcholine, a neurotoxic effect may occur. In other words, it is sensible to theorize that tauopathies progress via interaction of extracellular tau with M1 and M3 receptors on neurons leading to cytotoxic effects (Gómez-Ramos et al., 2009). Thus, blocking M1 and M3 receptors via receptor antagonists can prevent cytotoxic effects (Gómez-Ramos et al., 2008).

## Dynamin-driven endocytosis

Exogenous tau aggregates may be taken up via an active process attenuated by dynamin inhibition, supporting endocytosis-mediated internalization. Dynamin is a GTPase essential for multiple intracellular functions, including formation of vesicles from the cell membrane, endocytosis, and synaptic vesicle recycling among others (Kozlov, 1999). Evidence shows that tau aggregates colocalize with dextran and HeLa cells, hinting that internalized aggregates are transported in endosomal vesicles and passed through the endosomal pathway to lysosomes (Wu et al., 2013).

## Heparan sulfate proteoglycans–mediated macropinocytosis

Previous studies suggest that uptake of aggregated tau from the extracellular space depends on interaction with heparan sulfate proteoglycans (HSPGs; Holmes and Diamond, 2014). HSPGs are cell-surface macromolecules of heparan sulfate glycosaminoglycan chains covalently attached to a core protein. HSPGs are ubiquitously expressed in many cell types including neurons, and have been previously associated with dense core plaques, cerebrovascular amyloid, and NFT formation (van Horssen et al., 2001). Consistently, HSPGs have been implicated in amyloid as well as tau fibril formation *in vitro*, presumably facilitated by anionic moieties. Whether deposition of amyloid-b or tau is preceded by HSPGs or vice versa, it is clear that HSPGs play a role in the stabilization and uptake of these aggregates.

The recruitment of exogenous tau starts with binding HSPGs on the cell surface, stimulating macropinocytosis and bringing pathogenic “seeds” into the cell to guide trans-cellular propagation (Holmes et al., 2013). This uptake is necessary for intracellular seeding and was previously described for the prion protein uptake (Hooper, 2011). Even though the mechanism by which HSPGs mediate tau uptake is unknown, it seems to be confined to a specific “size” aggregate. Studies agree that small misfolded tau oligomers are readily taken up by neuronal cells (Wu et al., 2013; Mirbaha et al., 2015). However, regardless of the multiple “sizes” of tau aggregates that interact with the cell surface via HSPGs, it is likely that an assembly of at least three tau molecules is required to initiate endocytosis via HSPGs (Mirbaha et al., 2015). Interestingly, trimers were identified as the toxic tau aggregate at low nanomolar concentrations *in vitro* (Tian et al., 2013). Thus, tau oligomers may act as “seeds” inducing endogenous tau misfolding, suggesting a unifying mechanism for the propagation of protein amyloids (Mirbaha et al., 2015).

In other words, the HSPGs serve as a receptor for the cellular uptake of tau, a critical step similar to prion-like propagation. Basically, pathogenic tau aggregates use HSPGs to bind the cell surface of a neuron. This actively stimulates macropinocytosis, leading to propagation of aggregates between cells in culture and aggregate uptake *in vivo* (Holmes et al., 2013). Further, another study implied that exosomes depend on HSPGs for internalization (Christianson et al., 2013). As delineated above, exosomes are a distinct mechanism for propagation of misfolded tau.

## Annular protofibrils

A handful of proteins implicated in neurodegenerative diseases have been found to produce pore-like amyloid structures known as annular protofibrils (APFs). APFs are similar to pore-forming protein toxins in that their properties lead to membrane disruption. A recent study showed the existence of tau APFs in human brain samples from patients with PSP and LBD as well as in mice brain samples which overexpressed mutated tau. The study discovered that APFs form after tau oligomer formation and bypass higher NFT aggregate formation. The findings showed that APF formation relies on mutations in tau, phosphorylation levels, and cell type (Lasagna-Reeves et al., 2014). Hence, tau APFs may play a significant role in tauopathies by linking pore formation to cell death.

## Tau oligomers instigate mitochondrial damage

Oligomeric tau intermediates decrease cell viability (Flach et al., 2012). In aging, a protein involved in mitochondrial fission, dynamin-related protein 1 (DRP1), can bind tau abnormally, inducing neurodegeneration via mitochondrial dysfunction (Figure 2; DuBoff et al., 2012). Specifically, studies have shown reduced levels of mitochondrial proteins and activity in the brains of AD patients (Kim et al., 2001). One study showed diminished NADH-ubiquinone oxidoreductase (complex I) activity and injury to mitochondrial respiration and ATP synthesis (complex V) with age in P301L mice (David et al., 2005). Another study showed that expression of tau (truncated at Asp-421 to mimic caspase cleavage) caused mitochondrial dysfunction (Quintanilla et al., 2009).

Recently, data has shown that injected tau oligomers co-localize with the mitochondrial marker porin, suggesting a pathological relationship. In fact, tau oligomers might disrupt microtubule stability and trafficking, thus affecting organelle distribution. Mitochondria navigate long distances to provide for synaptic energy demand; therefore, inhibiting transport systems impairs energy production routes (Lasagna-Reeves et al., 2011).

Also, data shows low levels of complex I in brain hemispheres injected with tau oligomers when compared to brains injected with monomers or fibrils. This implies that alterations of complex I subunit mRNA, minimization in protein levels of complex I subunits (Kim et al., 2001), and other effects of mitochondrial damage (David et al., 2005) can be observed due to tau accumulation without the presence of NFT formation. Further, since tau oligomers hinder energy production through complex I, alterations in synaptically-localized mitochondria may result. However, recent data considering complex V levels suggested that tau oligomers do not implicate ATP synthesis initially. These results imply that tau oligomers initially affect complex I activity and may directly or indirectly disturb the later stage of complex V ATP synthesis (Lasagna-Reeves et al., 2011).

Mitochondrial damage can lead to activation of the apoptotic pathway. Hemispheres injected with tau oligomers were found to have increased levels of caspase-9 activation (Lasagna-Reeves et al., 2011). Suggestively, as tau oligomers concentrate at the mitochondrial membrane, cytochrome C is released, leading to caspase-9 activation via a complex with apoptotic-peptidase-activating-factor-1 (Apaf-1; Li et al., 1997). The relationship between cytochrome C and caspase-9 has been noted for other amyloidogenic proteins (Hashimoto et al., 1999; Simoneau et al., 2007). Moreover, caspase activation occurs before NFT formation, implying that a soluble tau entity may be the main toxic moiety (de Calignon et al., 2010).

Overall, evidence suggests that tau oligomers are the toxic entities in tau aggregation and that alteration of the mitochondrial membrane, minimization in complex I levels, and activation of the apoptotic-related caspase-9 can cause toxicity, impeding synaptic energy production (Lasagna-Reeves et al., 2011).

## Conclusion

Discovering the pathological role of tau oligomers within the brain along with related mechanisms of cellular tau oligomer secretion, propagation, and uptake will allow for a better understanding of tauopathies (Castillo-Carranza et al., 2013, 2014a; Gerson et al., 2014b). Further, mitochondrial dysfunction caused by internalized tau oligomers may play an important role in pathogenesis. Admittedly, little is known regarding cellular tau oligomer release. Yet with greater knowledge regarding disease pathogenesis, better therapeutic approaches can be generated. We hypothesize that preventing tau oligomers from cellular release and uptake via exosomal or ectosomal pathways will relieve some toxic effects induced by tau oligomers in tauopathies.

## Author contributions

SS, MG, and DC wrote the manuscript. DC and MG reviewed intellectual content. DC prepared the figures. SS, MG, DC, approved the final version of the manuscript.

### Conflict of interest statement

The authors declare that the research was conducted in the absence of any commercial or financial relationships that could be construed as a potential conflict of interest.
